# Operations for Otorrhoea

**Published:** 1908-03-28

**Authors:** 


### Otology.

### OPERATIONS FOR OTORRHŒA.

ACUTE MASTOIDITIS. PART II.

The first step in acute mastoiditis secondary to acute otitis media is to effect free tympanic drainage by myringotomy, *i.e.* by free incision of the tympanic membrane. If the pyrexia or pain or tenderness is uninfluenced by this treatment, in spite of purging and the use of local applications (heat or cold), then the mastoid cells should be opened. This measure is seldom resorted to until the second week following the onset of acute symptoms of middle-ear disease. It may, however, be necessary to open the mastoid earlier in acute otitis; for instance, when the mastoid swelling appears during the first five days of the disease and persists unrelieved by the above-mentioned treatment; or when severe toxic symptoms persist, as denoted by pyrexia, anorexia, and malaise, especially when accompanied by rigors; or when the local symptoms, pain and tenderness, increase day by day. It should be remembered, too, that meningeal symptoms sometimes set in early. Such would be an immediate indication for operative treatment.

The actual operation for acute mastoiditis so closely resembles the earlier stages of the complete radical tympano-mastoid operation, described fully in previous issues of The Hospital (October 9 and 26, 1907) that a brief *resumé* will suffice to indicate the chief points to which attention should be directed. To those unacquainted with the detailed anatomy of the variations of the mastoid, we would repeat that there are no landmarks whereby to indicate the nature of the mastoid one is about to open, whether access to the antrum is likely to be easily attained or whether an attempt to expose this cavity will be beset with extreme difficulty from start to finish. We cannot tell beforehand to what degree the lateral sinus, or the middle cranial fossa, encroaches on the field of operation, or will interfere with the opening up of the antrum or accessory cells.

The following special instruments are required: various gouges, a heavy metal hammer, sharp spoon curettes, bone forceps, seekers, and retractors. The post-aural incision should be made somewhat more freely than when performed for uncomplicated chronic otorrhœa, especially when the superficial mastoid tissues are infiltrated and swollen. The semicircular incision may be enlarged at the time found convenient by a horizontal incision at right angles from the middle of the posterior margin of the first. If an external fistula already exist the integuments should be incised through the fistula, and the track should be subsequently curetted and trimmed. The cortex of the mastoid is removed in successive layers with a broad shallow gouge, working upwards and forwards from the middle of the mastoid process towards the supra-meatal triangular depression. In cellular mastoids, the pus may well up as the first layer of bone is removed, whereas in dense mastoids, a considerable depth will be attained before the cells are opened and pus found. When diffusely infiltrated with pus the mastoid is readily excavated with the cross handled curette. At the same time great care is necessary to avoid wounding the dura mater, lateral sinus, facial nerve, and external semi-circular canal. It is necessary to eradicate every mucous-lined cell whether the seat of proliferous granulations or not. As the cavity is being enlarged it should be carefully inspected, and for this purpose must be kept free from blood and bone *débris*. It is extremely important to ascertain whether softened tracks in the bone lead to the dura mater, and, if so, these should be followed up with the object of exploring for an extra-dural abscess or for a dural fistula: the existence of these complications should never be overlooked.

In acute mastoiditis associated with recent primary acute otitis media, there is no need to open the attic or disturb the ossicles, provided distension of the tympanum proper be relieved by free incision of the membrane. In cases of chronic otorrhœa with acute mastoiditis the complete tympano-mastoid operation is, as a rule, performed.

Seeing there is some risk of meatal atresia after the simple mastoid operation, especially in cases in which the posterior osseous meatus has been freely removed, we may adopt the same plastic procedure as practised in the complete radical operation. Thus, a meato-conchal flap is cut to permit the posterior wall of the fibro-cartilaginous meatus being turned upwards against the roof of the mastoid cavity. This device permits the operation cavity to drain into the meatus, as well as through the post-aural wound. The cavity is tamponned with gauze through the enlarged meatus, and a tube is inserted in the heel of the post-aural incision. The dressings are then applied in the usual manner.

The patient should experience a feeling of relief immediately upon recovering from the operation. The earlier the stage of otitis media, the more likely is the otorrhœa to subside quickly. In those cases in which otorrhœa persists weeks after the simple operation, despite careful management, the cause may be found to lie in the tympanum or attic, and this would necessitate the removal of the outer attic wall together with the membrane and ossicles.

Upon the after treatment the results of the simple mastoid operation largely depend. Very careful supervision throughout the healing process is amply rewarded. The dressings must be changed daily, and the cavity irrigated with hydrogen peroxide (ten volumes per cent.), or with lysol 1 per cent., or with one in two thousand biniodide of mercury solution. Spongy granulations should be controlled by appropriate pressure with tampon, or should be destroyed with caustics or with the cautery. Sometimes the wound heals rapidly, sometimes it is very slow. The persistence of exuberant granulations or of fragments of crumbling bone should lead to the exploration of the cavity for a cause of the delay. A necrosed fragment of bone may be found, and after this has been removed the cavity should quickly heal and the otorrhœa should disappear.

